# Area-specific analysis of the distribution of hypothalamic neurons projecting to the rat ventral tegmental area, with special reference to the GABAergic and glutamatergic efferents

**DOI:** 10.3389/fnana.2015.00112

**Published:** 2015-09-04

**Authors:** Imre Kalló, Csilla S. Molnár, Sarolta Szöke, Csaba Fekete, Erik Hrabovszky, Zsolt Liposits

**Affiliations:** ^1^Laboratory of Endocrine Neurobiology, Institute of Experimental Medicine, Hungarian Academy of SciencesBudapest, Hungary; ^2^Faculty of Information Technology and Bionics, Pázmány Péter Catholic UniversityBudapest, Hungary; ^3^Laboratory of Integrative Neuroendocrinology, Institute of Experimental Medicine, Hungarian Academy of SciencesBudapest, Hungary; ^4^Department of Medicine, Division of Endocrinology, Diabetes and Metabolism, Tupper Research Institute, Tufts Medical CenterBoston, MA, USA

**Keywords:** preoptic area, hypothalamus, VTA, glutamate, GABA, retrograde labeling, *in situ* hybridization, immunohistochemistry

## Abstract

The ventral tegmental area (VTA) is a main regulator of reward and integrates a wide scale of hormonal and neuronal information. Feeding-, energy expenditure-, stress, adaptation- and reproduction-related hypothalamic signals are processed in the VTA and influence the reward processes. However, the neuroanatomical origin and chemical phenotype of neurons mediating these signals to the VTA have not been fully characterized. In this study we have systematically mapped hypothalamic neurons that project to the VTA using the retrograde tracer Choleratoxin B subunit (CTB) and analyzed their putative gamma-aminobutyric acid (GABA) and/or glutamate character with *in situ* hybridization in male rats. 23.93 ± 3.91% of hypothalamic neurons projecting to the VTA was found in preoptic and 76.27 ± 4.88% in anterior, tuberal and mammillary hypothalamic regions. Nearly half of the retrogradely-labeled neurons in the preoptic, and more than one third in the anterior, tuberal and mammillary hypothalamus appeared in medially located regions. The analyses of vesicular glutamate transporter 2 (VGLUT2) and glutamate decarboxylase 65 (GAD65) mRNA expression revealed both amino acid markers in different subsets of retrogradely-labeled hypothalamic neurons, typically with the predominance of the glutamatergic marker VGLUT2. About one tenth of CTB-IR neurons were GAD65-positive even in hypothalamic nuclei expressing primarily VGLUT2. Some regions were populated mostly by GAD65 mRNA-containing retrogradely-labeled neurons. These included the perifornical part of the lateral hypothalamus where 58.63 ± 19.04% of CTB-IR neurons were GABAergic. These results indicate that both the medial and lateral nuclear compartments of the hypothalamus provide substantial input to the VTA. Furthermore, colocalization studies revealed that these projections not only use glutamate but also GABA for neurotransmission. These GABAergic afferents may underlie important inhibitory mechanism to fine-tune the reward value of specific signals in the VTA.

## Introduction

Information processing through the reward pathway serves to promote survival of the individual, and consequently the species. Signals mediating basic homeostatic and social needs (i.e., hunger and thirst or territorial integrity and partner selection, respectively) reach the neuronal networks of the hypothalamus, which generate responses affecting feeding, energy expenditure, drinking, stress/adaptation and social interaction. Each response carries a reward value through interplay with the reward system of the brain, which appears ultimately in a behavior reflecting the level of reinforcement and motivation. Over the past decades, the central role of the ventral tegmental area (VTA) to regulate reward, motivation and reinforcement learning has been clarified and linked to its major dopaminergic cell population (Tzschentke and Schmidt, 2000; Gardner, 2005; Fields et al., 2007; Arias-Carrión et al., 2010). By using pathway tracing studies with classical neuroanatomical (Phillipson, 1979; Swanson, 1982; Holstege, 1987; Geisler and Zahm, 2005) and the recently elaborated genetic (Watabe-Uchida et al., 2012) approaches, the contributing principal neuronal connections have been identified. As part of these studies, hypothalamic inputs have been described. Phenotypic and functional characterization of some neurons projecting to the VTA has also been provided. Thus, subsets of neurons in the lateral hypothalamus containing orexin/hypocretin (Cason et al., 2010) or cocaine- and amphetamine-regulated transcript (CART)/melanin-concentrating hormone (MCH; Dallvechia-Adams et al., 2002) have been found to project to the VTA. Furthermore, neurotensin (NT) neurons projecting to the VTA have been identified in the medial (MPO) and lateral (LPO) preoptic area and the rostral lateral hypothalamus (rLH; Geisler and Zahm, 2006), and corticotropin-releasing factor (CRF) neurons in the paraventricular nucleus (Pa; Rodaros et al., 2007). In addition to these neuromodulatory pathways, tracing of afferents using the classical neurotransmitters glutamate and gamma-aminobutyric acid (GABA) to VTA neurons became also critically important. Of note, the basal activity of dopamine neurons is maintained in the VTA by a tonic excitatory N-methyl-D-aspartate (NMDA) receptor activation, counterbalanced by tonic inhibitory GABA receptor activation making disinhibition bursts and phasic dopamine release possible (Lobb et al., 2011; Morikawa and Paladini, 2011). Although previous studies detected vesicular glutamate transporter 2 (VGLUT2)-expressing glutamatergic afferents from the medial and lateral preoptic area (LPO), as well as from some nuclei of the medial and lateral hypothalamus to the VTA (Geisler et al., 2007; Geisler and Wise, 2008) and a GABAergic pathway from the MPO to the VTA (Tobiansky et al., 2013), the detailed map of hypothalamic neurons projecting to the VTA and the quantitative description of their glutamatergic or GABAergic character are not available.

In the present study, we have (i) systematically mapped hypothalamic nuclei that project to the VTA; and (ii) analyzed the GABA and/or glutamate characters of the identified hypothalamic afferent systems. Retrograde labeling of VTA-projecting hypothalamic neurons with cholera toxin B subunit (CTB) was combined with the isotopic *in situ* hybridization detection of glutamic acid decarboxylase 65 (GAD65) or VGLUT2 mRNA expression in male rats. The results indicate that the hypothalamus provide a substantial innervation to the VTA from a predominantly ipsilateral side and both from its medial and lateral nuclear compartments. In these communication channels connecting the hypothalamus to the VTA, glutamate and GABA are equally present and may critically contribute to the setting of VTA neuronal activity.

## Materials and Methods

### Animals

Adult male Wistar rats [300–350 g body weight (b.w.)] were used from a local breeding colony at the Medical Gene Technology Unit of the Institute of Experimental Medicine. They were housed under controlled lighting (12:12 light-dark cycle; lights on at 07:00 h) and temperature (22 ± 2°C), with access to food and water *ad libitum*. Experimental protocols were approved by the Animal Welfare Committee of the Institute of Experimental Medicine (No. A5769-01) and were carried out to meet the legal requirements of the European Community (Decree 86/609/EEC). During all surgeries, animals were under deep anesthesia by intraperitoneal administration of a cocktail of ketamine (25 mg/kg b.w.), xylavet (5 mg/kg b.w.) and pipolphen (2.5 mg/kg b.w.) made in saline.

### Retrograde Labeling of VTA-Projecting Hypothalamic Neurons with CTB

To label perikarya of hypothalamic neurons projecting to the VTA, the rats (*n* = 52) were given CTB (0.5% solution; #103, List Biological Laboratories, INC, Campbell, CA, USA) via unilateral iontophoresis (5 μA, 7 s on-off) into the VTA for 20 min. For targeting both mid-rostral and mid-caudal VTA regions, the following stereotaxic coordinates were used, respectively, with reference to the interaural planes; antero-posterior: +3.8 or +2.96 mm, medio-lateral: +0.6 mm, dorso-ventral: +2 or +1.8 mm (Paxinos and Watson, 2005).

### Tissue Collection and Processing

Ten days after tracer injections, the animals were perfused transcardially with 200 ml of 4% paraformaldehyde solution in 0.1 M sodium phosphate buffer (PBS, pH 7.4). Brains were removed, divided into a rostral and a caudal block with a coronal cut through the middle of the mammillary bodies (antero-posterior plane: interaural plane 4.8 mm; Paxinos and Watson, 2005). Both blocks were post-fixed at 4°C in the same solution for a few hours, soaked into 20% sucrose overnight, then, snap-frozen on powdered dry ice. Thirty [for single-label immunohistochemistry (IHC)]—or 20 [for combined IHC and *in situ* hybridization histochemistry (ISHH)]—μm-thick sections were cut in the coronal plane using a Leica SM 2000R freezing microtome (Leica Microsystems Nussloch GmbH, Nussloch, Germany) and transferred into six-well plates at a consecutive manner to produce six representative collections of sections from each brain. The free-floating sections were stored in antifreeze solution (30% ethylene glycol; 25% glycerol; 0.05 M phosphate buffer; pH 7.4) at −20°C until the histological staining.

### Single-Label Immunohistochemistry to Detect CTB

One out of the six groups of sections from each brain was processed for plotting the injection sites and mapping the distribution of the labeled cells. The floating sections underwent a series of pre-treatment steps, including permeabilization with Triton X-100 (0.5% in PBS, 20 min), blocking of endogenous peroxidase activity with H_2_O_2_ (0.5% in PBS, 20 min) and preventing non-specific antibody binding to the tissue with normal horse serum (2% in PBS, 10 min). The pre-treatment was followed by sequential incubations in goat anti-CTB serum (1:1,000; 24 h; RT; #703; List Biological Laboratories, INC, Campbell, CA, USA), in biotinylated donkey anti-goat IgG (1:500; 2 h; 705-065-147, Jackson Immunoresearch Laboratories, West Grove, PA, USA) and in avidin–biotin–peroxidase complex (ABC; 1:1,000; 2 h; PK-6100, Vector Laboratories). Between subsequent steps, the sections were rinsed thoroughly in PBS. The peroxidase reaction was developed in a solution containing 5% diaminobenzidine (DAB), 0.15% Ni-ammonium-sulfate and 0.006% H_2_O_2_ in Tris buffer (0.1 M; pH 7.6). The sections were then mounted onto gelatin-coated slides, counterstained with 1% toluidine blue, dehydrated through an ascending ethanol series, cleared in xylene and coverslipped with DPX (44581; Fluka Chemie AG, Buchs, Switzerland).

### Combined Detection of CTB and mRNAs for GAD65 or VGLUT2

#### Immunohistochemical Detection of CTB

To phenotype the retrogradely-labeled cells, immunohisto-chemical detection of CTB was combined with the *in situ* hybridization detection of GAD65 or VGLUT2 mRNA. Immunohistochemistry preceded the *in situ* hybridization steps, as preliminary results showed that the detection of CTB is considerably impaired following *in situ* hybridization. During immunohistochemistry, the tissue mRNAs were protected against enzymatic degradation by adding 1000 U/ml of heparin sodium salt to the immunohistochemical reagents and by using diethyl pyrocarbonate-pretreated and autoclaved 0.1 M PBS (pH 7.4) as a rinsing buffer between the incubation steps. The sections were treated with 0.5% H_2_O_2_ and 0.2% Triton X-100 (made in 0.1 M PBS) for 10 min then blocked against non-specific antibody binding with 2% bovine serum albumin (BSA; fraction V; Sigma) in PBS for 30 min and then, transferred into CTB antiserum (1:500) for 24 h. The primary antibodies were reacted with biotin-SP-anti-goat IgG (Jackson ImmunoResearch Laboratories; 1:500) and the ABC reagent for 1 h each. Then, biotin tyramide (diluted at 1:1,000 with TBS/0.002% H_2_O_2_ from a stock prepared in-house according to Adams (1992) and Kerstens et al. (1995) was deposited for 30 min on the peroxidase sites, as described previously (Hrabovszky et al., 2004a) and the sections processed for the *in situ* hybridization detection of GAD65 or VGLUT2 mRNA.

#### Preparation of GAD65 and VGLUT2 Probes

Preparation and use of an 879-base rat VGLUT2 cDNA (bases 522–1400 of VGLUT2 mRNA; NM_053427.1) have been described earlier (Hrabovszky et al., 2004b, 2005b). The rat GAD65–628 cDNA template (bases 315–944 of NM_012563.1) was kindly made available for these studies by Dr. Sandra L. Petersen (University of Massachusetts, Amherst, MA, USA; Hays et al., 2002). Complementary RNA probes were transcribed in the presence of ^35^S-UTP, as described elsewhere (Hrabovszky et al., 2004b). Specificity control experiments including the production and usage of sense probes and second antisense probes recognizing different sequences of target mRNAs have been described in these preceding papers.

#### *In situ* Hybridization Steps

Prior to hybridization, the sections were acetylated with 0.25% acetic anhydride in 0.9% NaCl/0.1 M triethanolamine (Sigma Chemical Company, St. Louis, MO, USA; pH 8.0) for 10 min, rinsed in standard saline citrate solution (2 × SSC; 1 × SSC = 0.15 M NaCl/0.015 M sodium citrate, pH 7.0) for 2 min, delipidated in 50, 70 and 50% acetone (5 min each) and then rinsed in 2 × SSC again. For hybridization, sections were transferred into microcentrifuge tubes filled with the hybridization solutions (Hrabovszky and Petersen, 2002). To reduce autoradiographic background, 1 M dithiothreitol was included in the hybridization buffer. The hybridization solution contained 40,000 cpm/μl radioisotopic probe and 10% dextran sulfate. Following hybridization at 52°C overnight, the non-specifically bound probes were digested with 20 μg/ml ribonuclease A (Sigma; dissolved in 0.5 M NaCl/10 mM Tris-HCl/1 mM EDTA; pH 7.8) for 60 min at 37°C and via a stringent treatment in 0.1 × SSC solution at 65°C for 90 min.

#### Visualization of CTB-immunoreactivity and GAD65 or VGLUT2 mRNA

Following posthybridization, the biotin tyramide deposits were reacted with streptavidin conjugated to the red fluorochrome Cy3 (Jackson; 1:1000; 1 h). Sections were then mounted onto silanized microscope slides, air-dried and processed for autoradiography. To visualize the isotopic signal, the sections were coated with Kodak NTB nuclear track emulsion (Kodak; Rochester, NY, USA; diluted 1:1 with distilled water) which was exposed for 4 weeks, then developed with Kodak processing chemicals. Sections underwent a counterstaining procedure with 10 μg/ml Hoechst (in 0.1 M PBS, pH 7.4, 1 min; Bisbenzimide, Sigma) to enable the distinction of hypothalamic nuclei. After a short rinse in PBS (1 min), the slides were dehydrated in 70, 96, and 100% ethanol (5 min each), cleared with xylene (2 × 5 min), and coverslipped with DPX.

### Analysis

#### Photomicrography

Single or mosaic digital photographs of each section were made by using a Zeiss AxioImager M1 microscope mounted with an AxioVision camera and the AxioVision 4.6 software (Zeiss, Göttingen, Germany) and saved as 8-bit TIFF files. The acquired image files were opened in photo-editing software (Adobe Photoshop CS5), composed (cropped, rotated) and adjusted for brightness, contrast, or color balance.

#### Plotting the Injection Sites

Sites of tracer deposition within the VTA or hypothalamus were plotted in low-power (10×) digital photographs (150 pixel/inch) of consecutive sections containing the hypothalamus and the midbrain. The position of plotted areas was evaluated with reference to counterstained neighboring nuclei and visible neuronal tracts. To map VTA-projecting neurons, sections were used from selected brains (*n* = 28) in which the tracer injection was precisely targeted and the focus did not exceed the boundaries of the VTA.

#### Mapping the Distribution of CTB-Labeled Neurons

CTB-labeled neurons were mapped in low-power (10×), high-resolution digital photographs (150 pixel/inch) of every sixth section of the hypothalamus. Peroxidase-stained specimens were photographed under bright-field illumination. Results of studies using combined immunofluorescence and autoradiography were first photographed with an epifluorescent filter set (excitation of 540–590 nm, band pass of 595 nm, and emission of 600–660 nm). Then, the autoradiographic image was captured using the dark-field illumination of silver grains. The fluorescent and autoradiographic images of the same section area were superimposed and processed as separate layers of Adobe Photoshop (PSD) files. Subdivisions of hypothalamic regions were determined by transposition of computerized standard series of rat brain atlas maps (Paxinos and Watson, 2005) in Adobe Photoshop multi-layered images. To adjust the atlas image to the sections produced, sections were counterstained either with 1% toluidine blue or 10 μg/ml Hoechst. In addition, other sets of sections were stained for hypothalamic peptides [i.e., oxytocin, vasopressin, NT, orexin, enkephalin, pro-opiomelanocortin (POMC), CART, thyrotropin releasing hormone (TRH), calcitonin gene related peptide (CGRP), CRH, and MCH] together with CTB (data not shown). The staining patterns obtained (beside the pattern generated by counterstaining, and the distribution of GAD65 and VGLUT2 transcripts) helped us delineate the hypothalamic subdivisions in our figures.

#### Quantification of VTA-Projecting GAD65- or VGLUT2 Expressing Neurons

Colocalization level of the immunofluorescent signal for CTB and the autoradiographic signal for GAD65 or VGLUT2 mRNA was analyzed in multi-layered digital images. For each region and brain, two to six photographs (representing coronal sections 120 μm apart from each other) were used for the analysis. A CTB-immunoreactive cell was considered double-labeled if the accumulation of the radioactive S^35^ hybridization signal (the silver grain cluster) overlapped largely with the somato-dendritic shape of the underlying stained cell and the density of the grains (area covered by silver grains/selected total area) was at least three times higher than in the background (the area fraction of silver grains in a surrounding background region), as determined with ImageJ software (public domain at http://rsb.info.nih.gov/ij/download/src/). For each area, the percentages of double-labeled cells were calculated and the data were expressed as the mean ± standard error.

## Results

### Cholera Toxin B Subunit Injections into the VTA Nuclear Complex

**Figure 1:**
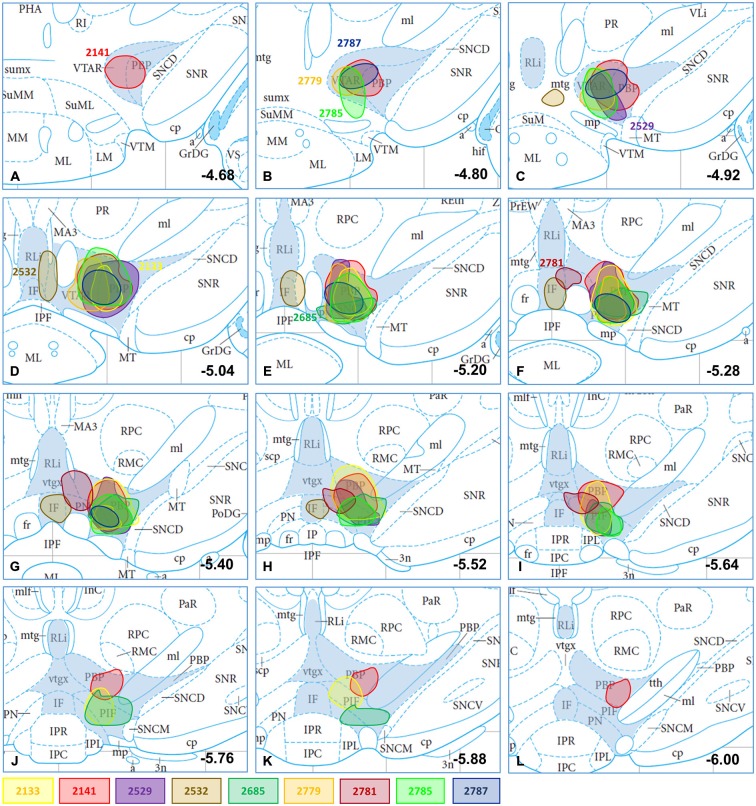
**CTB-immunoreactive injection sites plotted in mesencephalic section images; the location and dimension of selected injection sites (#2133, #2141, #2529, #2532, #2685, #2779, #2781, #2785, 2787) demonstrated with differently colored spots in atlas figures (Bregma levels from −4.68 to −6.00; A–L) according to Paxinos and Watson (2005)**. Subnuclei of the VTA (VTAR, PBP, PN, PIF, RLi, IF) are shaded in blue. The plots at different Bregma levels indicate the antero-posterior spread of the corresponding CTB deposit. The atlas figures modified were originally published in *The Rat Brain in Stereotaxic Coordinates: The New Coronal Set. Fifth edition, George Paxinos and Charles Watson, Copyright Elsevier (2005)*.

**Figure 2:**
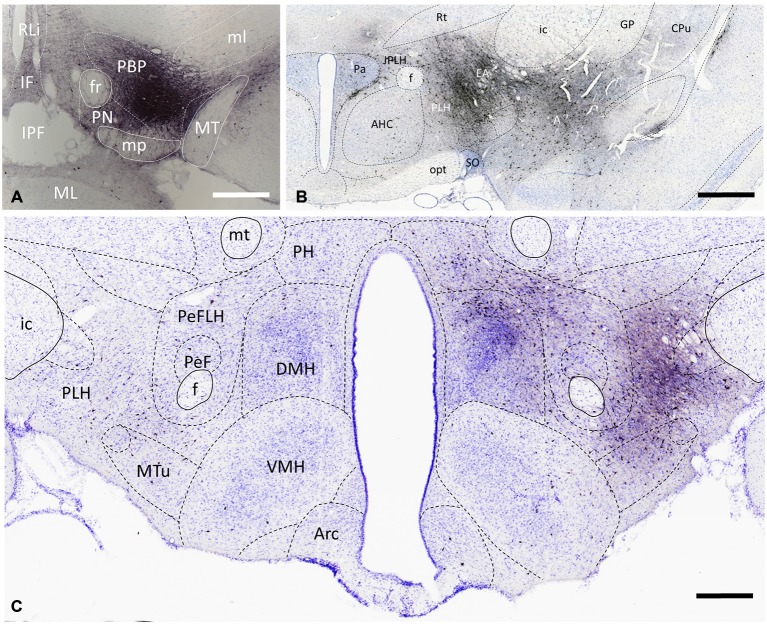
**Distribution of VTA-projecting neurons in the rat brain visualized by immunohistochemical detection of the retrograde tract tracer, cholera toxin B subunit (CTB)**. A representative CTB deposition (Case #2141) which is restricted to the parabrachial pigmented (PBP) nucleus of the VTA (at Bregma −5.2) is shown in **(A)**. Retrogradely labeled cells are present bilaterally with significantly higher number on the ipsilateral side, as shown in the area of the paraventricular **(B)** and dorsomedial **(C)** nuclei. CTB-immunoreactive cells are in the amygdala **(A)** and extended amygdala (EA) complexes, as well as in lateral, and medial hypothalamic areas. Boundaries of brain regions (dashed lines) correspond to those delineated in the rat brain atlas [75]. Sections were counterstained with Toluidine blue. Scale bars: 1000 μm for **(A,B)**, and 500 μm for **(C)**.

From 52 targeted CTB injections, 28 were restricted to the VTA. Tracer deposition was distributed variably among the different subnuclei of the VTA i.e., the rostral part of the VTA (VTAR), the parabrachial pigmented nucleus (PBP), the parainterfascicular nucleus (PIF), the paranigral nucleus (PN), the interfascicular nucleus (IF) and/or the rostral linear nucleus of the raphe (RLi; Oades and Halliday, 1987; Bourdy and Barrot, 2012). Nine different injections were selected to illustrate (Figure 1; Table 1) the results of the mapping and phenotypic characterization. These nine cases differed from each other in their antero-posterior (0.74 ± 0.1) and dorso-ventral (0.51 ± 0.04) dimensions as well as their location within the VTA. They were expanding between Bregma levels (−4.68) and (−6.00), with central sites located at (−5.04) and (−5.64; Table 1). With the exception of medial injection sites (e.g., #2532), CTB injections affected always the PBP and PN exhibiting variably sized deposition of the tracer (Table 1; case #2141 is illustrated with a photo in Figure 2A).

**Table 1:** **Characterization of the rostro-caudal spread of the different injection sites**.

							Bregma AP (mm)			RCDist (mm)
Case#	VTAR	PBP	PN	PIF	RLi	IF	B	C	E	
2133	+	++++++++	++	++++	−	−	−5.04	−5.28	−5.88	0.84
2141	++++	++++++++++++	+	++	−	−	−4.68	−5.20	−6.00	1.44
2529	++	++++++	++++	++	−	−	−4.92	−5.20	−5.64	0.72
2532	−	−	−	−	++	+++++	−4.92	−5.04	−5.52	0.60
2685	−	++++++	+++++++	++++	−	−	−5.20	−5.64	−5.88	0.68
2779	+++	++++	+++	−	−	−	−4.80	−5.20	−5.40	0.60
2781	−	+++	+++	++	+	+	−5.28	−5.28	−5.64	0.36
2785	+++	++++++	++++++	++	−	−	−4.80	−5.20	−5.64	0.84
2787	+++	++++++	++	−	−	−	−4.80	−5.28	−5.40	0.60

*The number of crosses refers to the number of sections containing CTB deposits in a given anatomical subdivision of VTA. B = the level of first appearance of the deposit, C = the level of the central portion of the deposit, E = the level of last appearance of the deposit, RCDist = rostro-caudal extension of the deposit*.

### Characterization of Retrograde Labeling in the Hypothalamus

In accordance with previous tracing studies (Phillipson, 1979; Swanson, 1982; Geisler and Zahm, 2005; Watabe-Uchida et al., 2012), CTB-IR neurons were detected in brain regions known to be part of the reward circuitry e.g., nucleus accumbens (NAc; not shown), prefrontal cortex (not shown), amygdala and extended amygdala (EA; Figure 2B). Within the hypothalamus, the retrogradely-labeled CTB-immunoreactive perikarya exhibited a predominantly ipsilateral distribution as illustrated in representative coronal sections at the level of the paraventricular nucleus (Figure 2B) and the dorso- and ventromedial nuclei (Figure 2C). From the retrogradely-labeled hypothalamic neurons the majority of descending fibers gathered in the medial forebrain bundle, resulting in an increasing rostro-caudal accumulation of CTB-immunoreactive axons also on the ipsilateral side (Figures 2B,C). Quantitative analysis of CTB-IR perikarya in nine different brains revealed similar distribution patterns. However, the number of retrogradely labeled cells differed from brain to brain. More than half of VTA-projecting neurons was found in anterior and mid hypothalamic regions (57.35 ± 5.04% of all counted CTB neurons); the preoptic areas and the posterior hypothalamic regions contained, respectively, 23.93 ± 3.91% and 18.92 ± 4.63% of the CTB-positive neurons. Although the lateral preoptic and lateral hypothalamic regions showed a more intense retrograde labeling in comparison with immunostained cellular and nuclear constituents of the medial hypothalamus (Figures 2B,C), overall nearly half (11.24 ± 2.52%) of the retrogradely-labeled neurons in the preoptic area, and more than one third (28.39 ± 3.22%) of them in the anterior, tuberal and mammillary hypothalamus appeared in medially located subdivisions; i.e., the MPA, the median preoptic, paraventricular, dorsomedial, ventromedial, mammillary nuclei and the SUM.

### Distribution and Transmitter Phenotype of Hypothalamic Neurons Projecting to VTA

#### Preoptic Input of the VTA

**Figure 3:**
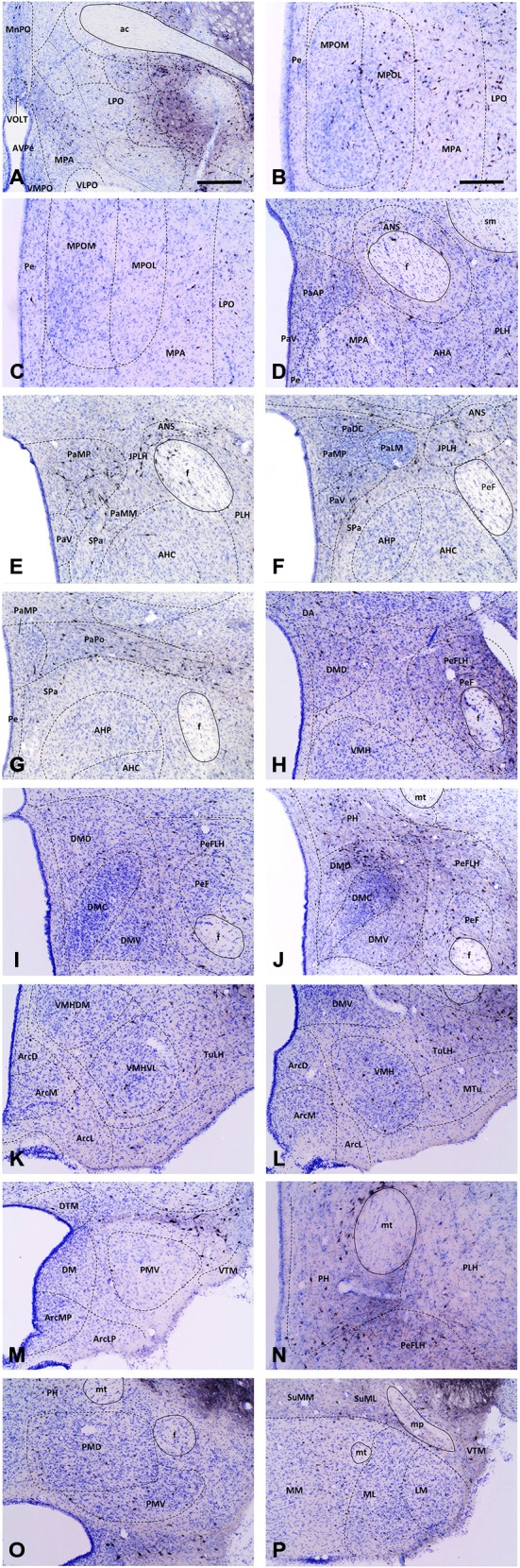
**Medium-power micrographs of representative coronal sections depicting main hypothalamic areas and nuclei of a CTB-injected brain (Case #2133)**. At certain rostro-caudal levels, the medial preoptic **(A–C)**, paraventricular **(D–G)**, dorsomedial **(H–J,L,M)** and posterior hypothalamic **(J,N,O)** nuclei exhibit high accumulation of VTA-projecting CTB-IR neurons. In contrast, the relatively few CTB-IR neurons in the ventromedial **(K,L)**, arcuate **(K–M)**, ventral and dorsal premammillary **(O)** and supramammillary and mammillary nuclei **(P)** are more-or-less evenly distributed through the rostro-caudal extent of these areas. Boundaries of brain regions (dashed lines) correspond to those delineated in the rat brain atlas. Scale bars: 500 μm in **(A)**, 250 μm in **(B–P)**.

The retrogradely labeled, CTB-IR neurons in the preoptic area were equally shared by the medial (11.24 ± 2.52%, calculated for all CTB positive neurons) and lateral (12.69 ± 1.67%) subdivisions. In the midline, relatively high numbers of CTB-IR neurons were found in the median preoptic nucleus (MnPO). In contrast, the vascular organ of the lamina terminalis (VOLT) contained retrogradely-labeled cells rarely (Table 2; Figure 3A). In medial preoptic regions, the majority of CTB-IR neurons were in the medial preoptic nucleus (MPO), predominantly in its lateral subdivision. A substantial number of labeled neurons was also detected in the MPA (Table 2; Figures 3B–D). No or very few CTB-IR neurons were found in the anteroventral preoptic nucleus (AVPO) or the ventromedial and ventrolateral preoptic nuclei (VMPO and VLPO; Table 2; Figure 3A). In these preoptic subdivisions, the VGLUT2 mRNA expressing, VTA-projecting neurons (VOLT: 44.44 ± 9.11%, MnPO: 51.43 ± 2.41%, AVPO: 55.77 ± 5.77%, MPA: 36.55 ± 5.85%, MPO: 45.78 ± 3.90%) predominated over the GAD65 mRNA containing neurons projecting to the VTA (VOLT: 3.12 ± 3.12%, MnPO: 9.30 ± 3.87%, AVPO: 5.00 ± 5.00, 18.26 ± 2.14, MPO: 14.17 ± 6.66%; Table 3). In lateral preoptic regions, intensely labeled CTB-IR neurons appeared in large number within the lateral preoptic area (LPO; Table 2; Figures 3A–C). The glutamate and GABA phenotypes were represented similarly (31.65 ± 3.47% vs. 29.90 ± 13.05%) in this brain region (Table 3).

**Table 2:** **Relative abundance of retrograde labeled cells resulting from six separate injections of CTB into the ventral tegmental area (VTA) of male rats**.

Hypothalamic regions	Case #2133	Case #2141	Case #2529	Case #2532	Case #2685	Case #2785
Vascular organ of the lamina terminalis (VOLT)	**+/−**	**+/−**	**+/−**	**+/−**	**+/−**	**+/−**
Medial preoptic area (MPA)	**+++**	**+++**	**+**	**++**	**+++**	**+++**
Median preoptic nucleus (MnPO)	**+++**	**++**	**+/−**	**++**	**++**	**+++**
Medial preoptic nucleus (MPO)	**++++**	**++++**	**+**	**++**	**++**	**++++**
*Medial part (MPOM)*	++	++	+/−	+	++	++
*Lateral part (MPOL)*	++	++	+	++	+	+++
Anteroventral preoptic nucleus (AVPO)	**+/−**	**+/−**	**−**	**−**	**+/−**	**+/−**
Ventromedial preoptic nucleus (VMPO)	**+/−**	**+/−**	**−**	**−**	**+/−**	**−**
Ventrolateral preoptic nucleus (VLPO)	**+/−**	**+/−**	**−**	**+/−**	**−**	**+/−**
Lateral preoptic area (LPO)	**+++**	**++++**	**++**	**++++**	**++++**	**++++**
Suprachiasmatic nucleus (SCh)	**+/−**	**−**	**−**	**−**	**+/−**	**+/−**
Supraoptic nucleus (SO)	**+/−**	**−**	**−**	**−**	**+/−**	**−**
Paraventricular hypothalamic nucleus (Pa)	**+++**	**+++**	**+**	**+/−**	**++**	**+++**
*Anterior parvicellular part* (PaAP)	++	+/−	−	−	+/−	+
*Medial magnocellular part* (PaMM)	++	++	−	+/−	+/−	+
*Medial parvicellular part* (PaMP)	++	+	+/−	−	+	++
*Dorsal cap* (PaDC)	+/−	+/−	+/−	−	+/−	+/−
*Lateral magnocellular part* (PaLM)	+	+	+/−	+/−	+/−	+
*Ventral part* (PaV)	+	++	+	+/−	+/−	+
*Posterior part* (PaPo)	++	+	+	+/−	+	++
Accessory neurosecretory nuclei (ANS)	**+**	**+/−**	**−**	**−**	**+/−**	**+/−**
Juxtaparaventricular part of lateral hypothalamus (JPLH)	**++**	**++**	**+/−**	**+**	**+/−**	**++**
Subparaventricular zone of the hypothalamus (SPa)	**++**	**++**	**+**	**+/−**	**+/−**	**++**
Anterior hypothalamic area (AH)	**+++**	**++**	**+**	**++**	**+**	**++**
*Anterior part* (AHA)	+	+	−	+/−	+/−	+
*Central part* (AHC)	++	+	+/−	+	+/−	+
*Posterior part* (AHP)	+	+/−	+/−	+/−	+/−	+
Lateroanterior hypothalamic nucleus (LA)	**+**	**+**	**−**	**+**	**+/−**	**+**
Arcuate nucleus (Arc)	**+**	**++**	**+**	**+**	**+**	**+**
Ventromedial hypothalamic nucleus (VMH)	**+++**	**+++**	**+**	**++**	**++**	**+++**
Peduncular part of lateral hypothalamus (PLH)	**+++++**	**+++++**	**+++**	**++++**	**++++**	**+++++**
Tuberal part of lateral hypothalamus (TuLH)	**+++**	**+++**	**++**	**++**	**+++**	**+++**
Dorsal hypothalamic area (DA)	**++**	**+++**	**+/−**	**+**	**+**	**++**
Dorsomedial hypothalamic nucleus (DM)	**+++**	**+++**	**+/−**	**++**	**++**	**+++**
*Dorsal part* (DMD)	+++	+++	+/−	++	++	+++
*Compact part* (DMC)	+	+	−	+/−	+/−	+
*Ventral part* (DMV)	+	+	+/−	+	+	+
Perifornical nucleus (PeF)	**++**	**++**	**+/−**	**+**	**+**	**++**
Perifornical part of lateral hypothalamus (PeFLH)	**++++**	**++++**	**+++**	**+++**	**+++**	**++++**
Medial tuberal nucleus (MTu)	**++**	**++**	**+/−**	**+**	**+**	**++**
Posterior hypothalamic nucleus (PH)	**+++**	**+++**	**++**	**+++**	**++**	**+++**
Posterior hypothalamic area (PHA)	**+**	**+**	**+/−**	**+/−**	**+**	**+**
Premammillary nucleus, ventral part (PMV)	**+**	**+**	**+/−**	**+**	**+**	**+**
Premammillary nucleus, dorsal part (PMD)	**+**	**+**	**+/−**	**++**	**+/−**	**+**
Medial mammillary nucleus, medial part (MM)	**+++**	**++**	**+/−**	**+++**	**+/−**	**++**
Medial mammillary nucleus, lateral part (ML)	**++**	**++**	**+/−**	**+++**	**−**	**++**
Lateral mammillary nucleus (LM)	**+**	**+**	**+/−**	**++**	**+/−**	**+**
Supramammillary nucleus, medial part (SuMM)	**++**	**+**	**+**	**++**	**+**	**++**
Supramammillary nucleus, lateral part (SuML)	**+++**	**++**	**+**	**++**	**+++**	**+++**
Dorsal tuberomammillary nucleus (DTM)	**+/−**	**+/−**	**+/−**	**+/−**	**+/−**	**+/−**
Ventral tuberomammillary nucleus (VTM)	**+**	**+/−**	**+/−**	**+**	**+/−**	**+/−**

*The CTB depositions appeared in different subnuclei of the VTA (between Bregma −4.68 and −6.00). The relative abundance of labeling is characterized using a semi-quantitative grading scale: −, 0; +/−, 1–10; +, 10–30; ++, 30–100; +++, 100–300; ++++, 300–1000; +++++, 1000–2000. The brain region hierarchy follows that of the Paxinos and Watson brain atlas*.

**Table 3:** **Colocalization percentages of GAD65 or VGLUT mRNA signals in VTA-projecting CTB-IR neurons in various hypothalamic areas**.

Hypothalamic regions	GAD (+) CTB (+) of all CTB (+) Mean ± SEM (%)	GAD (+) CTB (+) *n*_cells_	Only CTB (+) *n*_cells_	VGLUT2 (+) CTB (+) of all CTB (+) Mean ± SEM (%)	VGLUT2 (+) CTB (+) *n*_cells_	Only CTB (+) *n*_cells_
VOLT	3.12 ± 3.46	1	10	44.44 ± 9.11	19	18
MPA	18.26 ± 2.14	83	312	36.55 ± 5.85	148	198
MnPO	9.30 ± 3.87	13	81	51.43 ± 2.41	65	58
MPO	14.17 ± 6.64	83	312	45.78 ± 3.90	174	201
AVPO	5.00 ± 5.61	1	17	55.77 ± 5.77	9	7
LPO	29.90 ± 13.05	51	181	31.65 ± 3.47	88	205
Pa	10.28 ± 0.66	26	227	39.61 ± 2.45	144	216
JPLH	9.43 ± 3.41	23	141	40.65 ± 3.77	100	149
SPa	22.40 ± 7.88	17	43	24.05 ± 8.39	31	55
Arc	41.10 ± 17.44	11	13	41.67 ± 8.68	13	19
VMH	11.50 ± 6.87	9	89	59.24 ± 8.52	81	22
DMH	23.66 ± 14.24	42	66	43.63 ± 7.92	64	66
PLH	29.30 ± 4.87	330	767	33.83 ± 4.80	344	671
TuLH	40.24 ± 8.13	54	64	32.64 ± 12.75	30	79
PeFLH	58.63 ± 19.04	84	36	30.95 ± 17.97	36	54

*Data are presented as mean values of four animals ± SEM*.

#### Afferents of VTA Originating from Different Subdivisions of the Anterior Hypothalamus

**Figure 4:**
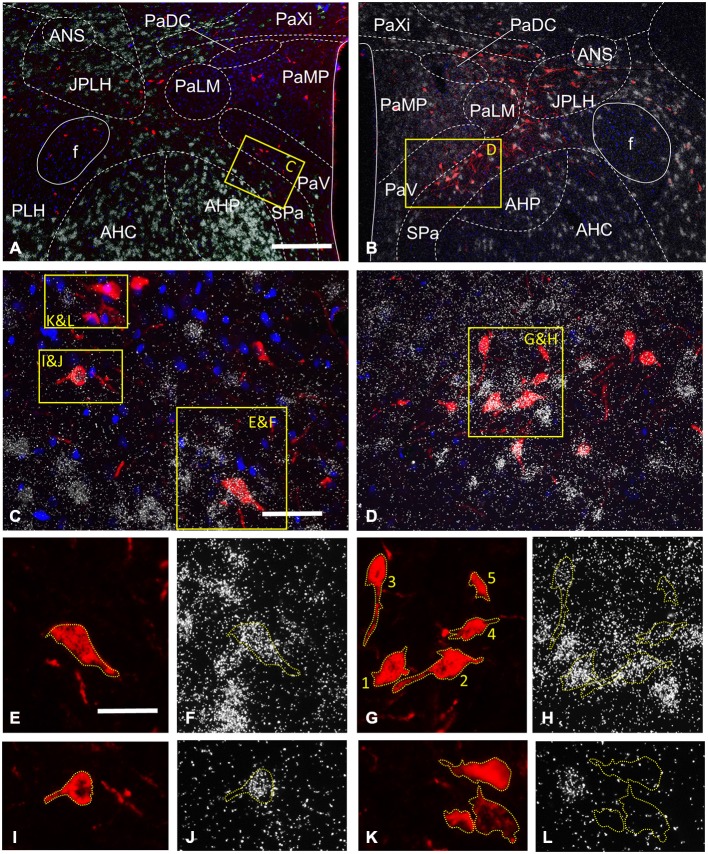
**Expression of the GABAergic (GAD 65 mRNA) and glutamatergic (VGLUT2 mRNA) markers by CTB-labeled hypothalamic neurons projecting to the VTA, as detected in two representative sections by the combined use of *in situ* hybridization (ISHH) and immunohistochemistry**. At the level of the paraventricular nucleus (Pa), CTB-IR neurons are detected in different subnuclei of the Pa, the juxtaparaventricular (JPLH) and peduncular (PLH) parts of the lateral hypothalamus and the subparaventricular zone of the hypothalamus (Spa; **A,B)**. Neurons with intense GAD65 mRNA expression are found in the anterior hypothalamic nuclei (AHP and AHC), the JPLH and SPa, and a few scattered cells also appear in the Pa **(A)**. A moderate vGLUT2 mRNA signal is present in the Pa, in contrast with the AHP, AHC, JPLH and SPa which contain neurons with high level of expression **(B)**. The yellow enframed areas in **(A,B)** are magnified to **(C,D)**, respectively. These images illustrate the SPa and the neighboring AHP and the ventral part of the Pa at medium power. The indexed yellow boxes are further magnified to single channel images, which show the signal either for CTB **(E,G,I,K)** or the mRNAs of GAD65 **(F,J,L)** or VGLUT2 **(H)**. The immunoreactive sites for CTB are in red due to the used fluorescent label **(E,G,I,K)**, whereas the autoradiographic signal marking mRNAs appear as white dots, due to the deposition of silver grains, were photographed at dark field illumination **(F,H,J,L)**. Yellow dashed lines are used—in both single channel images to highlight the position of the retrogradely-labeled cells under the silver grains. There are two CTB-positive cells expressing GAD65 mRNA in the SPA **(E,F,I,J)**, in contrast those in the PaV are not labeled for this marker of GABAergic neurons **(K,L)**. The autoradiographic signal varies from cell to cell, as exemplified by **(G,H)**; there are retrogradely-labeled cells showing strong autoradiographic signal for VGLUT2 mRNA in the SPa (cell number 1 and 2), a moderate signal in the PaV (cell number 3 and 4) and a signal, which fell below the threshold for considering as double-labeled (cell number 5). Scale bars: 200 μm for **(A,B)**, 50 μm for **(C,D)**, 25 μm for **(E,L)**.

The hypothalamic Pa contributed to the afferent supply of the VTA in a subnucleus-specific manner (Table 2). Moderate number of CTB-IR neurons were observed in the medial (PaMP; Figures 3E–G) and posterior (PaPO) parvocellular subdivisions (Figure 3G). Relatively few VTA-projecting neurons appeared in the ventral parvicellular (PaV; Figures 3D–F) and medial magnocellular (PaMM) subdivisions (Figure 3E) and only very few CTB-IR cells were located in the lateral magnocellular (PaLM) and the dorsal cap (PaDC) subdivisions (Figure 3F). In addition, moderate numbers of CTB-IR neurons were also observed in the immediate vicinity of the Pa, in the juxtaparaventricular part of the lateral hypothalamus (JPLH) and the subparaventricular zone of the hypothalamus (SPa; Figures 3E–G). The majority of the VTA-projecting neurons in the Pa (39.61 ± 2.45%) and JPLH (40.65 ± 3.77%) were identified as glutamatergic (Table 3; Figure 4). In contrast, nearly equal subsets of the CTB-immunopositive neurons were labeled for glutamatergic (24.05 ± 8.39%) and GABAergic (22.40 ± 7.88%) mRNA markers in the SPa region (Table 3; Figures 4E,F).

Very few CTB accumulating neurons were detected in the accessory neurosecretory nuclei (ANS; Table 2; Figures 3E,F), located at the level of the Pa above the fornix. The labeled neurons were virtually absent from the other major magnocellular neurosecretory nucleus, the supraoptic nucleus (SO), as well as from the suprachiasmatic nucleus (SCh). The anterior hypothalamic area contained only a few labeled cells. (Table 2; Figures 2B, 3D–G), contrasting the peduncular part of the lateral hypothalamus (PLH), which was heavily populated by a mixture of glutamatergic (29.30 ± 8.39%) and GABAergic (33.83 ± 4.8%), neurons projecting to VTA (Tables 2, 3; Figure 2B).

#### Projections to the VTA from the Tuberal Region of the Hypothalamus

The hypothalamic dorsomedial nucleus (DMH) contained a substantial number of CTB-IR neurons in its dorsal subdivision, mainly at caudal levels (Table 2; Figure 3J). In the compact and ventral subdivisions, very few or no CTB-IR neurons were detected (Figures 3I,J). In this nucleus, the VTA-projecting glutamatergic neurons predominated over the GABAergic ones (43.63% ± 7.92 vs. 23.66 ± 14.24%, Table 3).

The ventromedial nucleus (VMH) contained a moderate number of CTB-IR neurons (Table 2) which showed no preferential location in either the dorsomedial, the central or the ventrolateral subdivisions (Figures 2C, 3H,K,L). The majority of the labeled VMH neurons expressed VGLUT2 mRNA (59.24 ± 8.52%). The arcuate nucleus (Arc) contained only a few CTB-IR neurons (Figures 2C, 3K–M; Table 2). They consisted of a mixture of cells with glutamatergic (41.67 ± 8.68%) and GABAergic (41.10 ± 17.44%) phenotypes (Table 3).

In the mid-level of the hypothalamus, its lateral compartment was heavily populated by CTB-accumulating neurons. The labeled cells occurred in the perifornical (PeFLH) and tuberal (TuLH) subdivisions of the lateral hypothalamus, in the perifornical (PeF) and medial tuberal nuclei (MTu; Figures 2C, 3H–L). Contrasting more rostral regions, the perifornical (PeFLH) and the tuberal (TuLH) parts of the LH showed a predominance for GABAergic VTA-projecting neurons over those expressing the glutamatergic marker VGLUT2 mRNA (the frequency of VGLUT2 vs. GAD65 mRNA containing neurons was 32.64 ± 12.75% vs. 40.24 ± 8.13% in the TuLH and 30.95 ± 17.97 vs. 58.63 ± 19.04% in the PeFLH; Table 3).

#### Outflow from the Mammillary Region of the Hypothalamus to the VTA

Roughly one fifth of the VTA-projecting neurons were found in the caudal hypothalamic nuclei and regions (18.92 ± 4.63%). As elsewhere, CTB-labeled neurons consisted of mixed GABAergic and glutamatergic populations. Among the CTB-labeled cells, a substantial population of VTA-projecting neurons was found in the posterior hypothalamic nucleus (PH; Table 2; Figures 2C, 3J,N). CTB-IR neurons were also present in low numbers in the posterior hypothalamic area, and the dorsal and ventral parts of the premammillary nuclei (Table 2; Figures 3M,O), and nearly absent from the dorsal and ventral tuberomammillary nuclei (Table 2; Figures 3M,P). VTA-projecting neurons also occurred in the supramammillary nuclei (SuM) with the majority of the cells in the lateral part of this nucleus and the mammillary (MM) nuclei, where primarily the medial nucleus was labeled (Table 2; Figure 3P).

## Discussion

In the present report, we provide a detailed map of hypothalamic neurons that are wired to the VTA and demonstrate the differential co-localization of the employed tracer, CTB with GAD65 and VGLUT2 mRNAs in distinct nuclei and well-defined areas of the hypothalamus. The findings indicate that: (i) the hypothalamic input to VTA is bilateral with an ipsilateral predominance; (ii) in addition to the lateral hypothalamus, nuclei located in the medial segment of the hypothalamus also send intense neuronal projections to the VTA; (iii) composite nuclei provide subnucleus-specific, differential inputs; and (iv) a mixed glutamatergic and GABAergic population of preoptic/hypothalamic neurons communicates from both sides with the brainstem reward center, the VTA.

### Distribution of Neurons Projecting to the VTA in Hypothalamic Areas

The hypothalamic areas and nuclei showed a differential distribution of CTB immunoreactive neurons, exhibiting variations from abundantly to poorly populated regions. Similar distribution patterns were obvious in all injected animals suggesting the independence of the phenomenon from the size of CTB deposition. Thus, similarly to other investigators (Phillipson, 1979; Geisler and Zahm, 2005; Watabe-Uchida et al., 2012), we found a large number of VTA-projecting neurons in the lateral preoptic and lateral hypothalamic areas (LHs). In contrast, no or very few CTB-immunoreactive cells were observed in the ventrolateral preoptic, supraoptic and suprachiasmatic nuclei. Our mapping strategy of analyzing sections 180 μm apart from each other often revealed robust rostro-caudal differences as well in the number of CTB-IR cells within certain hypothalamic subdivisions i.e., the medial preoptic and the dorsomedial nuclei. This finding indicates the topographical segregation of neurons projecting to the VTA within the same nuclei. Thus, the majority of neurons projecting to the VTA were found in the rostral part of the medial preoptic area, which controls the appetitive component of the male sexual behavior (Balthazart and Ball, 2007), whereas only a few CTB-IR neurons were observed in the caudal half of the medial preoptic area and the adjacent rostral part of the anterior hypothalamic area, which have been implicated in attack against a male intruder (Veening et al., 2005) and in drinking regulation (Swanson et al., 1978). In the DMH, which is a site playing important physiological role in the circadian regulation of food intake and related anticipatory activation (Poulin and Timofeeva, 2008; Verhagen et al., 2011), the VTA-projecting neurons were found primarily in the dorsal and caudal subdivisions. It is currently unclear, whether the uneven distribution and different abundance of neurons projecting to the VTA in certain hypothalamic subdivisions reflect a function-dependent participation in direct communication with VTA.

To provide semi-quantitative measures for regional abundance and differences in the VTA-projecting neurons, we characterized the different hypothalamic areas and nuclear subdivisions using a 7-point scale i.e., characterizing brain regions exhibiting no or very few retrogradely-labeled neurons with—or ± signs, and those containing from ten to several thousand of CTB-positive cells with one to five plus signs. By using such approach, we could include in the analyses also hypothalamic nuclei containing relatively few neurons projecting to the VTA, and provide cumulative data for CTB-IR neurons occupying medially located subdivisions of the hypothalamus. The summary of our analyses confirmed previous observations, that proportionally fewer CTB-IR cells were on the contralateral than on the ipsilateral sides, but also produced some unexpected results i.e., half of the neurons projecting to the VTA in the preoptic region and one third in the anterior, tuberal and mammillary hypothalamus occurred in medial subdivisions.

Thus, significant subsets of neurons projecting to the VTA have been identified in the Pa, DM, and VMH. A few neurons were also observed to project to the VTA from the Arc. While we recognize that the low number of neurons traced from the VTA does not necessarily indicate a weak functional connectivity, it seems likely that the direct link between the primary target site of the metabolic signals, the Arc and the VTA is inferior to the indirect connections. Of note, the paucity of CTB-immunoreactive cells in the Arc seems to be in conflict with the results of AGRP- and β-endorphin-immunohistochemical studies showing relatively high density of immunoreactive fibers in the VTA (Finley et al., 1981; Dietrich et al., 2012). Since AGRP is exclusively synthetized by Arc NPY neurons (Chen et al., 1999) and the majority of β-Endorphin derive from Arc POMC neurons (Finley et al., 1981), we predicted that VTA injections of CTB would trace higher numbers of Arc neurons. The reason for not seeing more retrogradely labeled cells in the Arc might be that AGRP and β-endorphin-immunoreactive fibers pass through the VTA without establishing synaptic contact there. Another possibility is that only few AGRP and/or β-endorphin cells project to the VTA but they establish a particularly rich axonal arborization there. Further studies are required to clarify these issues.

In our present study, we found that hypothalamic neurons projecting to the VTA often occupy a position in the close proximity, but outside the borders, of major nuclei and pathways. Such accumulation of CTB-IR neurons was observed next to the hypothalamic paraventricular nucleus in the SPa and the JPLH and around the ventral and dorsal premammillary nuclei and in the caudal extension of the tuberal part of the lateral hypothalamus. This preferential distribution in the vicinity of certain hypothalamic nuclei suggests integrative functions for these neurons. By receiving limbic inputs (Ericson et al., 1991; Hahn and Swanson, 2010, 2012) and through connections with resident cells of the Pa, SO, DM, VMH, PMV, and PMD, these neurons may communicate stress- (Champagne et al., 1998), metabolic- (Park and Carr, 1998) and/or reproduction-related signals (Di Sebastiano and Coolen, 2012) to VTA to initiate motivation and reward. GABAergic neurons in the peri-Pa region (Roland and Sawchenko, 1993) are activated by both acute and chronic stress conditions, and respond to these conditions with, respectively, reduced or increased GAD65 mRNA expression (Bowers et al., 1998). An indirect pathway has already been described which originates from these “extranuclear” regions of the lateral hypothalamus (e.g., the juxtaparaventricular and juxtadorsomedial parts of the lateral hypothalamus) and mediates negative reward and aversive stimuli to the VTA (Matsumoto and Hikosaka, 2007; Hikosaka et al., 2008) via the lateral habenula (Hahn and Swanson, 2012). Very high numbers of CTB-positive neurons appeared in the PeFLH and the surrounded PeF, which encompasses discretely and differently defined lateral hypothalamic regions, including the juxtadorsomedial and the suprafornical regions, the connections of which have been extensively studied recently (Hahn and Swanson, 2012). It will be important to investigate how the predominantly GABAergic input from the PeFLH and PeF, and the mainly glutamatergic input from the JPLH contribute to the relation and integration of motivational or incentive value associated with the ingestive or defensive behaviors, respectively.

### VTA-Projecting Neurons Expressing the Glutamatergic Marker, VGLUT2 or the GABAergic Marker, GAD65 mRNA

Characterization of the transmitter phenotype of CTB-IR neurons in the hypothalamus revealed that neurons projecting to the VTA in all traced subdivisions consist of mixed glutamatergic (VGLUT2 mRNA expressing) and GABAergic (GAD65 mRNA expressing) populations. Even in hypothalamic nuclei known to express primarily VGLUT2 i.e., VMH or Pa, about one tenth of CTB-IR neurons were GAD65-positive. The proportion of glutamatergic and GABAergic neurons showed high variations within the different subdivisions. VGLUT2 mRNA signal was detected in 24–59% and GAD65 mRNA signal in 5–58% of CTB-IR neurons in the different regions and nuclei. Yet unknown subsets of the neurons projecting to the VTA secrete various peptides (Meister et al., 1988; Dallvechia-Adams et al., 2002; Hrabovszky and Liposits, 2008) that play important modulatory roles in the VTA (Korotkova et al., 2003, 2006; Geisler and Wise, 2008). Of note, GABA or glutamate may be present in certain peptidergic neurons, as it was concluded from ultrastructural data showing the presynaptic presence of small clear vesicles together with CRH (Tagliaferro and Morales, 2008), orexin/hypocretin (Balcita-Pedicino and Sesack, 2007) or CART (Dallvechia-Adams et al., 2002) in axon terminals establishing symmetric or asymmetric synapses. The presence of the same peptide in putative GABAergic and glutamatergic terminals raises the possibility that both VGLUT2 and GAD65 mRNA expressing neurons represent phenotypically diverse cell populations, and different peptide receptor subtypes (Wang et al., 2007; Boyson et al., 2011; Hwa et al., 2013) are involved in their signaling pathways.

The glutamatergic character of the VTA-projecting neurons was examined by *in situ* hybridization for VGLUT2 mRNA, which is the predominant vesicular glutamate transporter isoform in the hypothalamus (Ziegler et al., 2002). VGLUT2 mRNA expression could be detected in VTA-projecting neurons of all hypothalamic regions analyzed, suggesting that the double-labeled neurons contribute significantly to the subcortical glutamatergic innervation of the VTA. The percentage of double labeled neurons varied from 20 to 60%, with the highest values obtained for the VMH, AVPO, MnPO, MPO and VOLT. The lowest percentage of VGLUT2 expression in CTB-IR neurons was observed in the SPa. The co-localization level found in the current study is higher in most subdivisions than those previously reported for the whole preoptic area (~24%) or the anterior, tuberal and mammillary regions of the hypothalamus (~22%; Geisler et al., 2007); this may be due to differences in the applied tracing and histological methods and/or in the rat strains used. Glutamatergic synapses in the VTA undergo plastic changes, but the LTP-like phenomenon is only temporal in response to natural rewards, such as food. This is in contrast with the persistent changes evoked by drug abuse (Chen et al., 2008). To what extent the excitatory synapses of hypothalamic origin participate in the temporal changes induced by natural reward or in more persistent changes evoked by drug abuse is not known.

We selected a probe recognizing GAD65 mRNAs for the identification of GABAergic neurons, since most classes of GABA neurons in the CNS contain mRNAs for both GAD65 and GAD67 (Esclapez et al., 1993), and both are heavily present in the same hypothalamic regions. GAD65 expression could be detected in VTA-projecting neurons of all hypothalamic regions analyzed, suggesting that, besides glutamate, GABA also contributes significantly to the transmission of hypothalamic signals to VTA neurons. While GABAergic projections from the medial preoptic area to the VTA have already been identified (Tobiansky et al., 2013), our present study is the first to report an extensive GABAergic input to the VTA from diverse hypothalamic regions. We show relatively few GABAergic cells among the VTA-projecting neurons in the male MPA (18.26 ± 2.14%) and the MPO (14.17 ± 6.64%), in comparison with the high colocalization level (~68%) reported for female rats (Tobiansky et al., 2013). Some sex differences in the anatomical (Northcutt and Nguyen, 2014) and biochemical composition (Gardner, 2005; Ceylan-Isik et al., 2010; Lee et al., 2014) and in the electrophysiological properties (Melis et al., 2013) of the mesocorticostriatal system are well known. Thus, the discrepancy in co-localization levels might be due to sexually dimorphic projections from the MPA/MPO region to the VTA. Technical issues contributing to the differences, however, should also be taken into account. The highest percentage of GAD65 mRNA expression by VTA-projecting neurons was found in some LHs i.e., the perifornical (~58%), the tuberal (~40%) and the peduncular (~30%) parts, whereas the juxtaparaventricular part was an exception with its low colocalization percentage (~10%). The tuberal lateral hypothalamic neurons have a particular importance, since this region is associated with hunger recognition; damage to this area can cause reduced food intake (Williams et al., 2000; Konturek et al., 2005). The direct GABAergic pathways from the hypothalamic regions we identified in our present study supplement the GABAergic inputs arising from the rostro-medial tegmental nucleus (the tail of the VTA), which is thought to play an integrative role collecting information from the lateral habenula related to negative reward and aversion (Barrot et al., 2012; Bourdy and Barrot, 2012).

### Technical Considerations and Limitations

The percentages of GABAergic plus glutamatergic neurons projecting to the VTA in the different anatomical regions varied between 50% (e.g., VOLT, JPLH, SPa) to nearly 100% (Arc, PeFLH). This indicates that either a certain subset of VTA-projecting neurons does not express GAD65 or VGLUT2 mRNAs or in some cases the detection of these mRNAs fell below the sensitivity of our ISHH method. Although GAD65-expressing GABAergic and VGLUT2-expressing glutamatergic neurons exhibited distribution patterns complementary to one another both in the current, as well as our previous studies (Hrabovszky et al., 2012), analyses at single cell level revealed a subset of neurons being negative for these markers scattered within the predominantly GAD65- or VGLUT2 positive subdivisions of the hypothalamus. Of note, some of the hypothalamic GABAergic and glutamatergic neurons can only be identified by GAD67 (Esclapez et al., 1993) and VGLUT1 (Ziegler et al., 2002) expression, respectively. The absence of GABAergic and glutamatergic markers from peptidergic neurons of the hypothalamus is also possible. Accordingly, single-cell PCR data of Harthoorn et al. (2005) indicate that relatively large proportions of MCH or orexin neurons are devoid of both GABA and glutamate. The VTA consists of different cell types, but only the dopamine neurons are thought to mediate signal discrepancies between expected and actual rewards [33]. Ultrastructural studies on TH cells in samples labeled either for neuronal tracers injected to different parts of the brain, or neuropeptides synthetized in extra-VTA neurons suggest, that TH neurons receive inputs from both intrinsic (Omelchenko and Sesack, 2009) and extrinsic (Dallvechia-Adams et al., 2002; Omelchenko and Sesack, 2005, 2010; Balcita-Pedicino and Sesack, 2007; Tagliaferro and Morales, 2008) sources. Since both types of axon terminals establishing either symmetric or asymmetric synapses were detected on TH-IR neurons, it is very likely, that the GABAergic and glutamatergic neurons located in different subdivisions of the hypothalamus also contribute to the innervation of dopamine neurons. The identification of additional target cells in the VTA, require further studies. For example, the hypothalamic afferents of the functionally distinct caudal end of VTA containing primarily GABAergic neurons (the rostromedial tegmental nucleus, RMTg) have not been studied here. A recent article by Yetnikoff et al. (2015) revealed the hypothalamic afferent neurons of this region, which were not significantly different in distribution or number from those of the main VTA. Phenotyping of these hypothalamic afferents is an important goal of the oncoming experiments.

### Functional Considerations

The explored hypothalamic afferent systems of the VTA are thought to carry information about integrative physiological actions of the hypothalamus which ensure the proper operation and regulation of reproduction, feeding and energy expenditure, stress, adaptation, and sleep. These mechanisms maintain the homeostatic balance of the organism and are also supported by the reward system.

#### Feeding Regulation

Regarding the food intake, it is promoted by hunger, a homeostatic stimulus which activates the orexigenic NPY/AGRP neurons in the Arc and the orexin/hypocretin cells in the LH. Eating is terminated by satiety, which, in turn, activates the anorexigenic POMC/CART neurons in the Arc, as well as neurons of the ventromedial hypothalamic nucleus (VMH; Wu et al., 2014). Neurons projecting to the VTA from the Arc and VMH are relatively sparse. In contrast, large cell populations project to the VTA from the perifornical area (PeF), the LH and the dorsomedial hypothalamic nucleus (DM), regions activated in association with food reward processing (Hara et al., 2001; Harris and Aston-Jones, 2006; Tsujino and Sakurai, 2009), and in animals exhibiting food anticipatory behavior (Poulin and Timofeeva, 2008; Jiménez et al., 2013). Previous studies have already revealed the peptidergic phenotype of several neurons projecting to the VTA from these areas (for recent review, see Liu and Borgland, 2015). Thus, NT-containing neurons have been shown to innervate the VTA both directly (Geisler and Zahm, 2006; Kempadoo et al., 2013) and indirectly via orexin neurons and to mediate leptin’s effect for generating food reward (Leinninger et al., 2011; Opland et al., 2013). Besides contributing to the regulation of the homeostatic and hedonistic components of food intake (Hurley and Johnson, 2014; Valdivia et al., 2014), the orexin-containing neurons have been reported to play important roles in reward processing and addiction (Aston-Jones et al., 2010; Cason et al., 2010). Finally, MCH-containing neurons have also been shown to connect the homeostatic and reward systems (Sherwood et al., 2012). Of note, the primary classical neurotransmitter in orexin neurons is glutamate, whereas the MCH and the NT neurons use mainly GABA (Meister et al., 1989; Meister, 2007). The specific way of using the classical neurotransmitters and neuropeptides within the neuronal circuits operating during homeostatic control of energy balance vs. hedonistic food intake is far from being fully understood.

#### Regulation of Drinking

Drinking has been shown to increase dopamine release in the VTA, suggesting a reward value of rehydration for the animals (Yoshida et al., 1992). Both acute and chronic dehydration activate neurons in osmosensitive areas i.e., the supraoptic (SON) and paraventricular nuclei (PVN), VOLT, the MnPO and the subfornical organ (SFO). cFos activation is silenced by rehydration in each of these regions, as shown on transgenic rats expressing cFos-fluorescent reporter fusion proteins (Yoshimura et al., 2013). Dopamine release when the activity of osmosensitive areas is silenced suggests a reduced inhibitory input to VTA dopaminergic neurons from these areas. Considering that the SON and the magnocellular subdivision of the Pa contain no or very few VTA-projecting neurons, and most VTA-projecting neurons in the Pa, VOLT, and MnPO are glutamatergic, it is very likely that they terminate on VTA’s local inhibitory neurons. This postulation, however, needs to be confirmed.

#### Stress and Adaption

Interactions between stress and the mesocorticolimbic dopamine system have been suggested by multiple behavioral and electrophysiological studies showing stressor-induced dopamine release in the prefrontal cortex (Abercrombie et al., 1989), in the NAc shell (Kalivas et al., 1987) and in the basolateral amygdala (Inglis and Moghaddam, 1999). Glutamate is thought to be a key mediator of stressor signals in the VTA (Wang et al., 2005, 2012), the effect of which is enhanced by various neuromodulators, including CRH (Wise and Morales, 2010), and hypocretin/orexin (Wang et al., 2009). Consistent with the colocalization of VGLUT2 and CRH (Hrabovszky et al., 2005c) or hypocretin/orexin (Rosin et al., 2003) in subsets of paraventricular or lateral hypothalamic neurons, respectively, it is likely that the current tracing study identified relatively large numbers of VTA-projecting VGLUT2/CRH and VGLUT2/hypocretin/orexin neurons at these sites.

#### Social Interaction

There is abundant evidence for the rewarding feature of social interactions. Anticipation of sexual activity and mating in males and females induces cFos activation and dopamine release in the NAc (Damsma et al., 1992; Pfaus et al., 1995; Bradley and Meisel, 2001; Jenkins and Becker, 2003). Dopamine has also been implicated in the expression of maternal behaviors. Pup presentation to a lactating dam increases levels of dopamine (Hansen et al., 1993) and cFos activity (Fleming et al., 1994) in the NAc, whereas removal of dopamine fibers or pharmacological blockage of dopamine receptors in the NAc impairs maternal behaviors (Hansen, 1994; Keer and Stern, 1999). The above results strongly suggest that mesolimbic activity plays a critical role in the rewarding component of sexual and maternal behaviors. The relatively high number of neurons projecting to the VTA in preoptic and hypothalamic centers of sexual and maternal behaviors (i.e., the MPO and VMH), raises the possibility that these nuclei are the source of reward signals in this context.

It is well known that aggressive/dominance behavior induces cFos activation in the VTA (Smith et al., 1997; Miczek et al., 2011; Gil et al., 2013; Wang et al., 2013), as well as in the hypothalamic attack area involving the lateroanterior hypothalamic and the anterior hypothalamic nuclei, the retrochiasmatic area, the ventrolateral part of the VMH, and the tuber cinereum area (dorsolateral to the ventromedial nucleus; Hrabovszky et al., 2005a). This area appears to be different from brain structures involved in defense against predators, which consists of the anterior hypothalamic nucleus, dorsomedial part of the VMH, and the dorsal premammillary nucleus (Motta et al., 2009), brain regions which are highly interconnected (Thompson and Swanson, 2010). Most of these areas contain only a few VTA-projecting neurons, with the exception of the tuberal part of the lateral hypothalamus, which was well-populated by a mixture of GABAergic and glutamatergic neurons in our present study.

**Figure 5:**
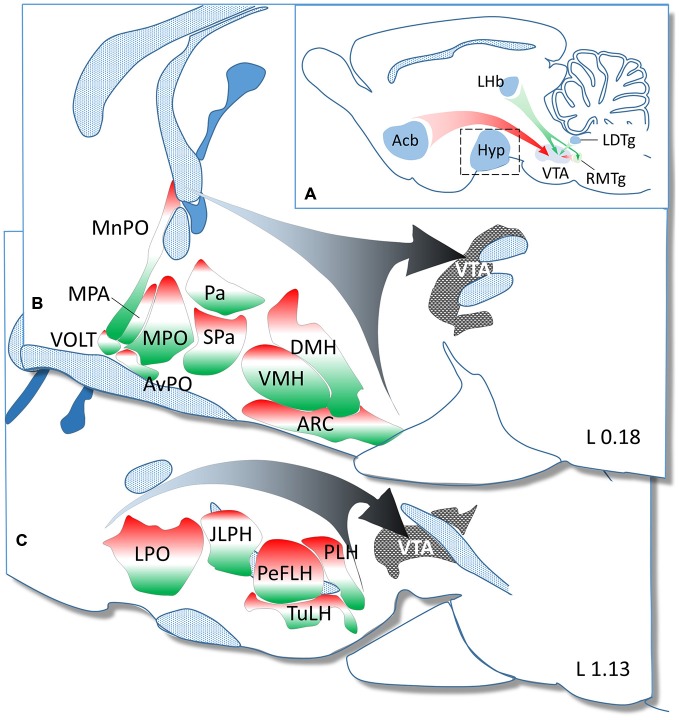
**Summary diagram to demonstrate the GABAergic and glutamatergic phenotype in neurons projecting from hypothalamic regions to the ventral tegmental area (VTA). (A)** In addition to the major GABAergic (from the NAc and RMtg) and glutamatergic (from the LHb and LDtg) inputs, the hypothalamus (enframed) also represents a significant source of GABAergic and glutamatergic afferents to the VTA. The hypothalamic nuclei and regions demonstrated in two paramedian sagittal sections (**B** = L 0.18 and **C** = L 1.13 mm) are color-coded to illustrate the relative ratio of GAD65 (red) and VGLUT2 (green) expressing neurons. The white color shows the relative ratio of neurons, which either do not express these markers or express them below the detection threshold of ISHH.

In conclusion, results of our present study provide detailed information about medial and lateral hypothalamic regions that provide neuronal input to the VTA. Among these cells, the proportion of glutamatergic and GABAergic neurons showed regional variation (Figure 5), with a predominance of glutamatergic neurons in most hypothalamic nuclei including the VMH, MnPO or Pa, and GABAergic neurons in lateral hypothalamic subdivisions involving the PeFLH and TuLH. In addition, neurons, which fail to express the GABAergic and glutamatergic markers or expressing them at low levels are also numerous in most hypothalamic regions. This suggests, that neurons using other neurotransmitters and/or neuropeptides may also contribute significantly to the hypothalamic innervation of the VTA.

## Conflict of Interest Statement

The authors declare that the research was conducted in the absence of any commercial or financial relationships that could be construed as a potential conflict of interest.

## Abbreviations

Acb: accumbens nucleus
AH: anterior hypothalamic area
AHA: anterior part
AHC: central part
AHP: posterior part
Arc: arcuate nucleus
AVPO: anteroventral preoptic nucleus
ANS: accessory neurosecretory nuclei
DA: dorsal hypothalamic area
DM: dorsomedial hypothalamic nucleus
DMD: dorsal part
DMC: compact part
DMV: ventral part
DTM: dorsal tuberomammillary nucleus
IF: interfascicular nucleus
JPLH: juxtaparaventricular part of lateral hypothalamus
LA: lateroanterior hypothalamic nucleus
LDTg: laterodorsal tegmental nucleus
LHb: lateral habenular nucleus
LM: lateral mammillary nucleus
LPO: lateral preoptic area
ML: medial mammillary nucleus, lateral part
MM: medial mammillary nucleus, medial part
MnPO: median preoptic nucleus
MPA: medial preoptic area
MPO: medial preoptic nucleus
MPOM: medial part
MPOL: lateral part
MTu: medial tuberal nucleus
PA: paraventricular hypothalamic nucleus
PaAP: anterior parvicellular part
PaMM: medial magnocellular part
PaMP: medial parvicellular part
PaDC: dorsal cap
PaLM: lateral magnocellular part
PaV: ventral part
PaPO: posterior part
PaXi: paraxiphoid nucleus
PBP: parabrachial pigmented nucleus
PeF: perifornical nucleus
PeFLH: perifornical part of lateral hypothalamus
PIF: parainterfascicular nucleus
PLH: peduncular part of lateral hypothalamus
PH: posterior hypothalamic nucleus
PHA: posterior hypothalamic area
PHD: posterior hypothalamic area, dorsal part
PMV: premammillary nucleus, ventral part
PMD: premammillary nucleus, dorsal part
PN: paranigral nucleus
RLi: rostral linear nucleus of the raphe
RMTg: rostromedial tegmental nucleus
SCh: suprachiasmatic nucleus
SO: supraoptic nucleus
SPa: subparaventricular zone of the hypothalamus
SuMM: supramammillary nucleus, medial part
SuML: supramammillary nucleus, lateral part
TuLH: tuberal part of lateral hypothalamus
VLPO: ventrolateral preoptic nucleus
VMH: ventromedial hypothalamic nucleus
VMPO: ventromedial preoptic nucleus
VOLT: vascular organ of the lamina terminalis
VTA: ventral tegmental area
VTM: ventral tuberomammillary nucleus.
